# Matrix Metalloproteinase-9 Testing of Golden Rice Cookies With Piper Crocatum Active Extract for Preventing Foot Ulcers in Patients With Diabetes: Protocol for a Randomized Controlled Trial

**DOI:** 10.2196/49940

**Published:** 2024-02-29

**Authors:** Andina Setyawati, Ariyanti Saleh, Takdir Tahir, Saldy Yusuf, Syahrul Syahrul, Aminuddin Aminuddin, Muhammad Raihan, Nuurhidayat Jafar, Hasyrul Hamzah, Nur Arfian

**Affiliations:** 1 Medical Surgical Nursing Department Faculty of Nursing Hasanuddin University Makassar Indonesia; 2 Department of Psychiatric Nursing, Faculty of Nursing Hasanuddin University Makassar Indonesia; 3 Department of Nutrition Medicine Faculty Hasanuddin University Makassar Indonesia; 4 Faculty of Pharmacy Hasanuddin University Makassar Indonesia; 5 Community Health Nursing Department Nursing Faculty Hasanuddin University Makassar Indonesia; 6 Faculty of Pharmacy Universitas Muhammadiyah Kalimantan Timur Kalimantan Timur Indonesia; 7 Department of Anatomy Faculty of Medicine, Public Health and Nursing Universitas Gadjah Mada Yogyakarta Indonesia

**Keywords:** diabetic foot ulcer, prevention, diabetic neuropathy, cookies, food supplement, study protocol

## Abstract

**Background:**

Diabetic foot ulcers (DFUs) present a formidable challenge to both patients and health care systems. DFUs significantly reduce the quality of life for patients, prolong hospital stays, and are the cause of approximately 70,000 lower limb amputations across the globe annually. Prevention of DFUs primarily involves the optimization of blood sugar levels and the effective management of complications, particularly peripheral neuropathy. Golden Rice has been proven to lower blood sugar levels due to its beta-carotene content, and Piper crocatum (*P. crocatum*) has been found to be effective in reducing the risk factors of DFUs through biomolecular regulation because of its polyphenol content.

**Objective:**

The principal objective of this study is to identify the efficacy of *P. crocatum*–enriched cookies, with Golden Rice as their primary ingredient, in preventing DFUs. The evaluation will center on their impact on the expression of matrix metalloproteinase-9 (MMP-9), a pivotal factor in the development of DFUs.

**Methods:**

This study is an experimental clinical research that follows the randomized controlled trial method and uses a single-blind design. The participants in the study are outpatients from primary health centers in Makassar, Indonesia, who have been diagnosed with diabetes mellitus. The sample for the study will be randomly selected and subsequently categorized into 2 groups: the intervention group and the control group. The intervention group consumes *P. crocatum*–enriched Golden Rice cookies, while the control group receives cookies without these additives. The participants from both groups will consume their respective cookies (packaged identically) twice a day for 14 days. The cookies will be prepared according to a modified recipe with an emphasis on low glucose content, resulting in 51 calories per cookie, comprising 1% carbohydrates, 6% fat, 4% cholesterol, and 4% fiber, excluding gluten, sugar, and salt. They will be baked at 158°C for 20 minutes. The process involves the addition of 20% Golden Rice and 10% *P. crocatum* ethanol extract, both prepared via maceration with 96% ethanol. The dependent variable in this study is the expression of gelatinases matrix metalloproteinase, to be assessed at 2 distinct time points—preintervention (pretest) and postintervention (posttest)—with the evaluation conducted through the western blotting method.

**Results:**

The recruitment and testing phase started in January 2024. The study is scheduled to be completed by the end of March 2024. Data analysis will commence in April 2024, and the publication of the results is anticipated in the same year (2024). The study will report on the changes in primary data, encompassing gelatinases matrix metalloproteinase, as well as secondary data, including the ankle-brachial index, neuropathy score, and random blood glucose level.

**Conclusions:**

The findings of this trial are expected to significantly impact the selection of strategies by health care practitioners to enhance diabetes self-management, particularly in the domain of therapeutic snacking, for patients diagnosed with diabetes mellitus.

**Trial Registration:**

Thai Clinical Trials Registry TCTR20230502001; https://www.thaiclinicaltrials.org/show/TCTR20230502001

**International Registered Report Identifier (IRRID):**

PRR1-10.2196/49940

## Introduction

Diabetic foot ulcer (DFU) is a major problem in patients with diabetes, affecting 15% of the diabetic population. DFUs significantly reduce the quality of life for patients, prolong hospital stays, and are the cause of approximately 70,000 lower limb amputations across the globe each year [1]. Diabetic foot disease is among the top 10 medical conditions in terms of burden, and it is estimated that up to 34% of individuals with diabetes will experience DFU at some point in their lives [2]. Preventing DFUs and amputations is of utmost importance to alleviate this significant burden on patients, health care systems, and society as a whole [3].

Peripheral neuropathy, the most prevalent type of diabetic neuropathy, primarily affects the nerves in the extremities, especially the feet. It accounts for 6.4% of all complications related to diabetes [4]. This condition predominantly disrupts sensory function, leading to gradual numbness, which increases the susceptibility to developing ulcers due to external injuries. It is crucial to identify these risk factors to enhance the effectiveness of preventive measures for DFUs [5]. Treating diabetic neuropathy presents a significant clinical hurdle. According to some studies, the gelatinase matrix metalloproteinase 9 (MMP-9) has been found to play a vital role in the demyelination of axons and the development of diabetic neuropathy in rodents [6,7]. As gelatinase MMP-9 plays a pivotal role in the initial onset of neuropathy, it is considered crucial in the progression of diabetic neuropathy and could potentially be targeted for treatment purposes [6,7].

Elevated levels of blood sugar and insulin resistance in patients with type 2 diabetes further contribute to the generation of reactive oxygen species (ROS), which intensifies the complications associated with this disease [8]. The activity of vascular MMP-9 is heightened in individuals with diabetes mellitus, partly due to increased production by vascular endothelial cells. Additionally, the activity of ROS plays a significant role in this process [9]. The high levels of ROS and MMP-9 have been found to be associated with glycemic index and diabetes complications [10].

Different types of diabetic-friendly cookies and breads are already in circulation, such as cassava bread, flax meal powder, and grape seed oil bread. However, their purpose is solely to limit glucose intake rather than directly affecting insulin levels. In contrast, noncookie products made with Golden Rice as the main ingredient contain beta-carotene, which can stimulate insulin production in the body by boosting the beta pancreas and decreasing the glycemic index [11]. However, the reduction in glycemic index needs to be supported by the biomolecular environment as an effort to prevent DFUs. The suitable herbal ingredient for combination in these cookies is *Piper crocatum (P. crocatum*). The oral consumption of *P. crocatum*, belonging to the Piperaceae family, has been proven safe among the population in Indonesia [12].

The use of *P. crocatum* has been documented to exhibit numerous therapeutic activities, such as anti-inflammatory effects. It has shown to reduce the expression of tumor necrosis factor alpha, nitric oxide, and interleukin-1β in a liver injury model using rats [13]. In addition, *P. crocatum* also exhibits wound healing activities by increasing the expression of superoxide dismutase 1, alpha-smooth muscle actin, and E-cadherin, and reducing p53 in hyperglycemic fibroblasts [14]. It has antibacterial properties when used topically [15]. *P. crocatum* acts as an antioxidant by reducing the expression of ROS and increasing glutathione peroxidase and cytochrome P450 2E1 [16]. It also demonstrates antihyperglycemic effects when administered orally [17].

Based on relevant studies on *P. crocatum* and its various mechanisms, *P. crocatum* can be used as a base ingredient for cookie-based therapies for the prevention of DFU. If the reduction of MMP-9 is recognized as a beneficial therapeutic target for DFU treatment [18], MMP-9 would also be a favorable target for DFU prevention. This is because the activity and expression of vascular MMP-9 in patients with diabetes mellitus are inherently elevated [19]. Furthermore, the gelatinase MMP-9 plays a crucial role in the pathogenesis of diabetic neuropathy through axonal demyelination, which is one of the risk factors for DFU [6]. Therefore, this study aims to identify the effectiveness of *P. crocatum* cookies with Golden Rice as the main ingredient for preventing DFUs through the regulation mechanism of MMP-9.

## Methods

### Study Design

**Figure 1:**
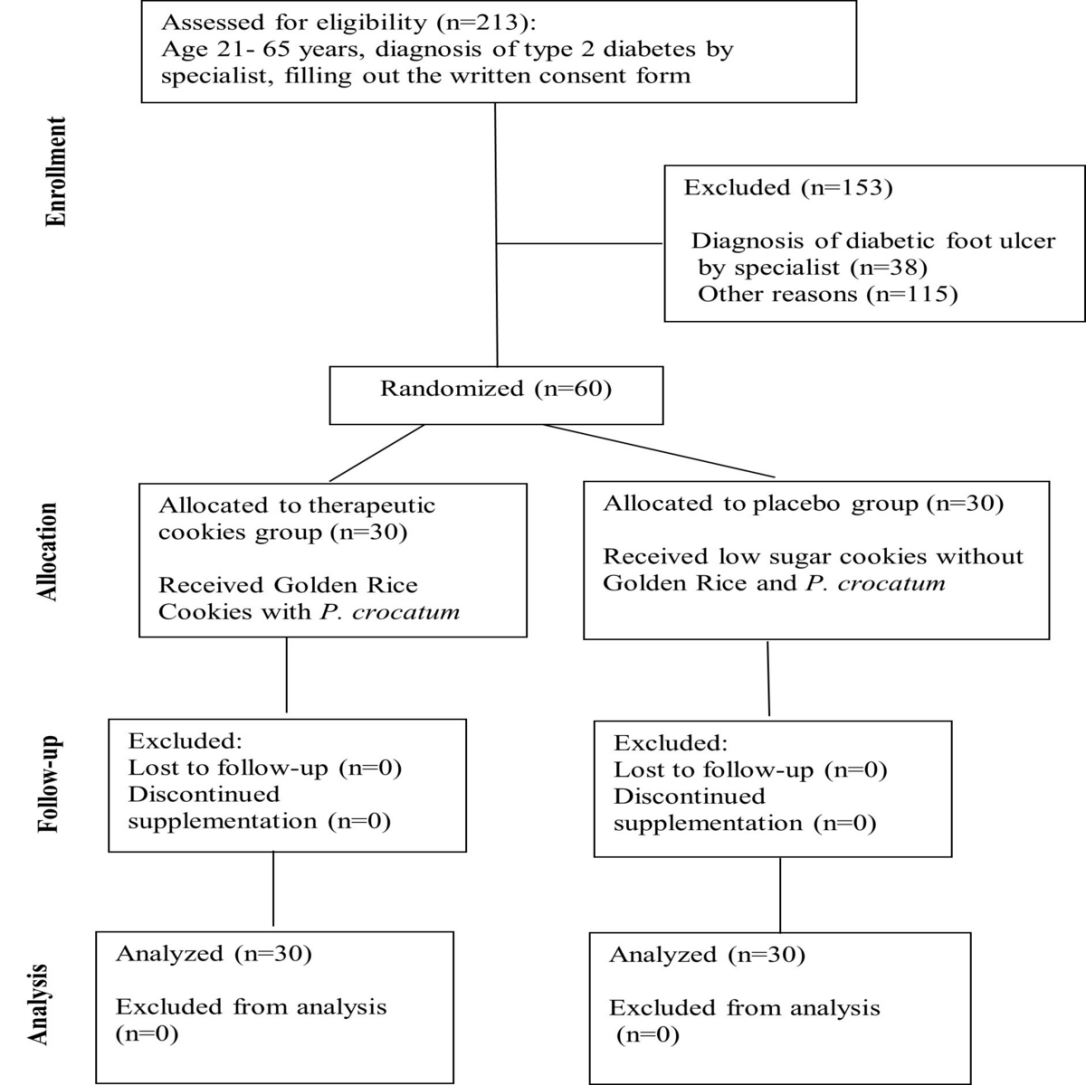
The CONSORT (Consolidated Standards of Reporting Trials) flowchart for participant recruitment and progress through a randomized controlled trial investigating the efficacy of Golden Rice cookies with Piper crocatum (*P. crocatum*) active extract in preventing foot ulcers among patients with diabetes at a public health center in Makassar, Indonesia, from November to December 2023.

This randomized controlled trial will be conducted at a public health center in Makassar, Indonesia, following a 2-arm, parallel-group, randomized, double-blind, placebo-controlled design. The study protocol was developed in accordance with the SPIRIT (Standard Protocol Items: Recommendations for Interventional Trials) 2013 checklist (Multimedia Appendix 1). Figure 1 shows the flowchart detailing participant enrollment, allocation, intervention, and assessment, and Table 1 presents the participant timeline.

**Table 1:** Participant timeline in a randomized controlled trial investigating the efficacy of Golden Rice cookies with Piper crocatum active extract in preventing foot ulcers among patients with diabetes at a public health center in Makassar, Indonesia, from November to December 2023.

Activities		Enrollment	Allocation	Postallocation			Close-out
		Week 1	Week 2	Week 3	Week 4	Week 5	
**Enrollment**							
	Eligibility screening	✓					
	Informed consent	✓					
	Randomization		✓				
	Allocation		✓				
	Patients training		✓				
**Intervention**							
	Supplementation			✓	✓		
	Compliance			✓	✓		
	Adverse events			✓	✓		
**Assessment**							
	Demographics		✓			✓	
	Clinical information		✓			✓	
	Neuropathy score		✓			✓	
	Ankle-brachial index		✓			✓	
	Random blood glucose		✓			✓	
	Supplement checklist					✓	

### Outcomes

The main focus of this study, or the primary outcome, is determining the level of MMP-9. The secondary outcomes include examining neuropathy scores and ankle-brachial index (ABI) as well as assessing random blood glucose.

### Randomization and Blinding

After obtaining informed consent and discussing the research objectives, a total of 60 eligible participants will be divided into 2 equal groups. Block randomization with a 1:1 allocation ratio will be used. An assistant will conduct the block randomization, ensuring that both the investigators and participants remain blinded to the intervention allocation. The sequence of the blocks will be generated using a random numbers table. All participants will be randomly assigned to either the intervention group or the placebo group. The manufacturer responsible for preparing the supplements will be requested to label the cans containing either therapeutic cookies or placebos with a unique code.

### Sample Size Calculation

With a significance level (type 1 error) of 5% and a statistical power of 90%, the sample size for each study group will be calculated to be 25, using a 2-sided *t* test, taking into account the changes in MMP-9 values as one of the primary outcomes [20]. We will clarify that the block lengths in our randomization process will be constant. To account for an estimated attrition rate of approximately 20% during the study, the final sample size will be increased to 30 participants in each group. The estimation of the sample size is calculated using the following formula:



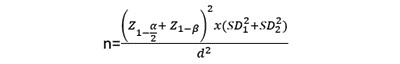



In this formula, α=5%, 1-β=90%, SD1=2.7, SD2=2.7, *d*=2.5.

### Cookies Production

Cookies will be created by following the formula provided by Olawoye et al [21], with some modifications. They will be made with a low glucose ingredient composition, resulting in each cookie containing 51 calories, with a composition of 1% carbohydrates, 6% fat, 4% cholesterol, and 4% fiber, free from gluten, sugar, and salt. Following the optimal conditions for producing gluten-free cookies for patients with diabetes, the cookies are baked at a temperature of 158°C for 20 minutes. The procedure is modified by incorporating 20% Golden Rice extract and 10% *P. crocatum* ethanol extract. Each extract will be prepared using the maceration method with 96% ethanol, and its yield will be calculated. The specific details of the *P. crocatum* extraction procedure are mentioned in a previous study [14].

### Intervention

A total of 60 eligible participants diagnosed with type 2 diabetes will be assigned randomly to either the therapeutic cookies group (n=30) or the placebo group (n=30). Over 14 weeks, participants in the intervention group will take therapeutic cookies (20 g) twice daily, while participants in the control group will take identical placebo cookies. The therapeutic cookies will be provided by Evelyn Food Supplement company, chosen for their reputation for providing high-quality, standardized ingredients, which is essential for the integrity of the study. Both therapeutic and placebo cookies will be indistinguishable in terms of weight, size, shape, taste, color, and odor. The placebo cookies are also produced by Evelyn Food Supplement company. The choice of company was made to maintain the highest research standards and to ensure that there are no conflicts of interest, financial support, or other compensation for the researchers.

The trial will consist of 3 study visits: before the intervention, 1 week after the intervention, and at the end of the intervention period. Study participants will receive instructions on how to use their supplements and will be followed up with phone calls every 3 days throughout the study. Compliance will be assessed by evaluating the number of unused supplements returned by each individual, with the remaining cookies counted to determine total supplement intake. Additionally, participants will be asked to maintain a reporting diary to document any adverse events experienced following the consumption of therapeutic cookies. If any adverse events are attributed to the consumption of therapeutic cookies, participants will be instructed to discontinue taking the supplements and will be promptly referred to a specialist for appropriate treatment. The study participants will not receive any specific dietary recommendations or dietary regimens.

### Blood Sample Collection

Blood samples for the identification of MMP-9 will be collected from each participant at a specific time point in the morning, following an overnight fast. The samples will be drawn at approximately 8 AM to ensure consistency and minimize potential variations in MMP-9 levels due to diurnal rhythms. This fasting state collection will aim to provide a baseline measurement of MMP-9 levels. Additionally, blood samples will be collected both before and after the intervention involving the administration of cookies to assess potential changes in MMP-9 levels in response to the intervention.

### Measures and Measurements

#### Demographic Questions

Participants will be requested to provide information about their gender, age, marital status, the medication and herbs currently being consumed, profession, educational level, distance from the place of residence to the nearest health care service, residence in a city or rural area, duration of having diabetes, and any other diseases or illnesses experienced.

#### Primary Outcome

A blood sample of 5 mL will be collected, and the samples will be centrifuged at 3000 rpm for 5 minutes to separate the serum samples before and after the intervention. The levels of MMP-9 will be determined using western blotting in triplicate. Proteins will be extracted using M-PER Mammalian Protein Extraction Reagent (Thermo; catalog number 87785) according to the manufacturer’s instructions. Protein quantification will be performed using Pierce 660 nm protein assay reagent (Thermo; catalog number 22660). The proteins will be separated on a 10% sodium dodecyl sulfate-polyacrylamide gel electrophoresis and transferred to a polyvinylidene fluoride membrane (Immun-Blot, Bio-Rad). Then, the membrane will be incubated with specific primary antibodies, including anti–MMP-9 (catalog number EP1254; anti-rabbit; 1:500 dilution) and anti–β-actin (catalog number ab8227; anti-rabbit; 1:1000 dilution). Blocking will be performed using 5% skim milk in Tris-Buffered Saline with Tween 20, followed by incubation with the appropriate secondary antibody. The protein bands will be visualized using enhanced chemiluminescence Prime Western Blotting Detection Reagents (GE Health Care; catalog number RPN2232), and the blots will be captured using a Gel Doc machine (Geldoc Syngene Gbox Seri Chemi xrq) [22].

#### Secondary Outcomes

The secondary outcomes include examining neuropathy score, ABI, and random blood glucose. The neuropathy score will be assessed following a previous study [23], using a 10-g Semmes-Weinstein Monofilament. During the monofilament testing, the participants will be instructed to lie flat on the examination table. To ensure that they could not see their feet, a standard monofilament (5.07/10-gram Semmes-Weinstein nylon monofilament) will be used to lightly apply pressure to three specific areas of the participants’ feet: (1) the underside of the first metatarsal head, (2) the underside of the fifth metatarsal head, and (3) the top surface between the first and second metatarsals. The monofilament will be positioned on the foot’s surface, forming a perpendicular angle with the skin. The pressure will be gradually intensified until the filament is bent, indicating the application of a predetermined level of pressure. The sites will be examined in a random order, rather than following a specific sequence. If the examinee provided the correct response at any site during the test, further testing at that site was not necessary. However, if the examinee could not correctly identify the interval in which the stimulus was applied, the test would be repeated at that site up to 2 times, aiming to obtain 2 similar correct responses. A site will be considered sensate if either the first response is correct or 2 out of 3 tests yield a correct response. On the other hand, a site will be classified as insensate if there are 2 incorrect responses and 2 “unable to determine” responses or 1 incorrect response and 1 “unable to determine” response. The presence of an insensate area at any of the 3 sites indicates a positive result for peripheral neuropathy in the monofilament test [24,25].

The ABI will be determined by measuring the systolic blood pressure in the arm and leg after a 10-minute rest in a supine position, with both arms and legs straight and relaxed [26]. Manual cuffs will be used for all blood pressure measurements, and the appropriate cuff size will be selected based on the arm circumference determined during screening. The same cuff size will be used for the lower leg, and a straight wrapping technique will be employed. The arm blood pressure will be measured using a sphygmomanometer along with an 8 MHz Doppler device to detect pulses. A single measurement will be taken at each of the 6 sites in the following sequence: left arm, left ankle (dorsalis pedis and posterior tibialis), right arm, and right ankle. The right ABI will be calculated by dividing the higher-pressure measurement from the right ankle (dorsalis pedis or posterior tibialis) by the higher brachial pressure (right or left side). The left ABI will be calculated using a similar method. The lower ratio from either side will be considered as the participant’s ABI [27].

To evaluate the random blood glucose levels, blood samples will be obtained from the capillary of the index finger for each patient. Subsequently, the Accu-Chek Active glucometer, a device manufactured in Germany, will be used to measure the samples, and the recorded values will be documented [28].

### Statistical Analyses

The demographic characteristics of all participants will be summarized using descriptive statistics, such as means (SDs), frequencies, and ranges, which will be presented in a tabular form. Statistical analysis of the data will be performed using the SPSS statistical software (version 2, SPSS Inc). *P*<.05 will be considered statistically significant. The normality of the data will be assessed using the Kolmogorov-Smirnov test. Numerical variables will be presented as mean (SD) or median (IQR) values, while categorical variables will be presented as frequency (percentages). To determine differences in numerical variables between the therapeutic cookies and placebo groups, independent sample *t* tests or Mann-Whitney *U* tests will be used. The same tests will also be used to compare alterations in outcome variables between the 2 groups. For within-group comparisons, paired-sample *t* tests or the nonparametric Wilcoxon test will be used. To account for the effects of confounding factors, a general linear model will be applied. In addition, analysis of covariance will be used to account for the effects of confounding factors when appropriate.

### Ethical Considerations

Prior to the commencement of this study, ethical review and approval were obtained from the local ethical committee of the Public Health Faculty of Universitas Hasanuddin, Makassar, Indonesia (approval number 10973/UN4.14.1/TP.01.02/2023). This study adheres to all applicable ethical guidelines and regulations governing research involving human subjects.

Informed consent was obtained orally from all participants involved in the primary and data collection. In the case of secondary analyses of research data, the original informed consent, as approved by the institutional review board, explicitly allowed for secondary analyses without the need for additional consent. Participants were informed that their data would be used for research purposes beyond the initial study.

The privacy and confidentiality of participants in this research were rigorously protected. All data collected were anonymized or deidentified to ensure the confidentiality of participants. Any personal identifying information was removed, and data were stored securely to prevent unauthorized access.

Participants received compensation in the form of monetary compensation for their time and participation. The compensation amount was set at IDR 100,000/week (US $7-8/week) and was disclosed to participants during the informed consent process.

The study has been registered on the Thai Clinical Trials Registry website (TCTR20230502001). Any changes to the study protocol will be communicated to the Trials journal.

## Results

The recruitment and testing phase started in January 2024. The study is scheduled to be completed by the end of March 2024. Data analysis will commence in April 2024, and the publication of the results is anticipated in the same year (2024).

## Discussion

### Expected Outcomes

This study focuses on the significant issue of DFUs in patients with diabetes and the importance of preventing DFUs and associated amputations. DFUs affect approximately 6.4% of the global diabetic population [29], reduce quality of life, prolong hospital stays, and lead to a considerable number of lower limb amputations globally each year [30]. Notably, peripheral neuropathy, the most prevalent type of diabetic neuropathy, plays a critical role in the development of DFUs due to its impact on sensory function and increased susceptibility to ulcers [31].

In the realm of diabetic neuropathy, MMP-9 is considered crucial in its progression and holds a potential target for treatment purposes [32]. Furthermore, it is important to acknowledge that elevated blood sugar levels and insulin resistance in diabetes contribute to the generation of ROS, intensifying the complications associated with the disease [33]. The activity of MMP-9 is heightened in individuals with diabetes, partially due to increased production by vascular endothelial cells and the influence of ROS [33].

MMP-9 plays a role in increasing tissue inflammation, which is one of the contributing factors to diabetic neuropathy [32]. By reducing MMP-9 activity, we can decrease inflammation that harms peripheral nerves. MMP-9 can damage blood vessel walls, leading to impaired blood circulation to nerves [34]. Reducing vascular damage can enhance blood flow to nerves, aiding in the prevention of neuropathy. Additionally, MMP-9 can harm the extracellular matrix surrounding nerve cells. If MMP-9 is reduced, the extracellular matrix may be better preserved, offering improved protection against nerve damage. Excessive MMP-9 activity has been linked to apoptosis or nerve cell death [35]. By reducing MMP-9 activity, we can help safeguard nerve cells from death, which contributes to neuropathy. Furthermore, MMP-9 can contribute to nerve degeneration and loss of nerve function. Lowering MMP-9 can assist in slowing down or halting this degenerative process [36].

The main interest of this study is the concept of diabetic-friendly cookies, which can stimulate insulin production in the body by boosting the beta pancreas and decreasing the glycemic index through the mechanism of MMP-9 reduction. To further support the reduction in glycemic index and prevent DFU, the suitable herbal ingredients for combination in these cookies are Golden Rice and *P. crocatum*. They are known for their safety and therapeutic activities, such as anti-inflammatory effects, wound healing activities, antibacterial properties, antioxidant effects, and antihyperglycemic effects. The oral consumption of *P. crocatum* and Golden Rice extracts has been proven to be safe in humans. A functional beverage containing *P. crocatum* was developed, demonstrating significant antioxidant and antidiabetic properties, and it was acceptable by consumers [37]. A recent study has also identified the use of *P. crocatum* as a healthy food and drink option for reducing blood sugar levels in patients with diabetes mellitus [38].

The reduction of MMP-9 as the study objective may not be sufficient to completely prevent diabetic neuropathy, as this disease is highly complex and involves numerous factors. However, reducing MMP-9 can be a potentially helpful step in mitigating the risk and impact of diabetic neuropathy, particularly when combined with other appropriate treatment approaches.

This study explores the biological mechanisms underlying diabetic neuropathy and the potential role of MMP-9, providing valuable insights into the pathophysiology of DFUs and potential therapeutic targets. The safety and acceptance of *P. crocatum* and Golden Rice extracts in humans, as demonstrated in previous studies, support the feasibility of incorporating these ingredients into dietary interventions to prevent DFUs.

Although this study delves into biological mechanisms and potential therapeutic interventions, the translation of these laboratory findings into clinical applications may require further research and clinical trials. The study may have limitations regarding the generalizability of its findings. It is essential to acknowledge that dietary interventions may have varying effects on individuals with different demographics, comorbidities, and genetic factors. The study duration is also considered a limitation, as it does not cover a long-term assessment of the effects of cookies and herbal ingredients.

### Conclusions

In conclusion, the findings of this study hold the potential to significantly influence the strategies used by health care practitioners in enhancing diabetes self-management, particularly in the domain of therapeutic snacking, for patients diagnosed with diabetes mellitus. Considering the substantial burden of DFUs on patients, health care systems, and society, it is crucial to focus on preventing DFUs and amputations. Exploring potential therapeutic targets like MMP-9 and using natural ingredients like *P. crocatum* in combination with other diabetes-friendly products may provide promising avenues for preventing DFUs and improving the outcomes for patients with diabetes. Further research and clinical trials would be necessary to validate the efficacy and safety of such interventions. These insights can inform the selection of strategies for health care practitioners to enhance diabetes self-management, thereby benefiting patients with diabetes mellitus. 

## Appendix

Multimedia Appendix 1 SPIRIT Checklist.

Multimedia Appendix 2 Consort checklist.

## Abbreviations

ABI: ankle-brachial index
DFU: diabetic foot ulcer
MMP-9: matrix metalloproteinase-9
*Piper crocatum*: *P. crocatum*
ROS: reactive oxygen species
SPIRIT: Standard Protocol Items: Recommendations for Interventional Trials

## Data Availability

The data sets generated and analyzed during this study are not publicly available due to patient confidentiality concerns but are available from the corresponding author upon reasonable request. All requests will be subject to review and approval on a case-by-case basis. We are dedicated to making the data available, where possible, while ensuring the protection of participants’ confidentiality and privacy.
